# Re: Enigmatic morphological traits in human teeth from early bronze age

**DOI:** 10.1007/s10266-016-0285-y

**Published:** 2016-12-08

**Authors:** Agnieszka Przystańska, Mariusz Glapiński, Tomasz Kulczyk

**Affiliations:** 1 Department of Anatomy, Poznań University of Medical Sciences, Poznań, Poland; 2 Department of Oral Rehabilitation, Poznań University of Medical Sciences, Poznań, Poland; 3 Department of Oral Radiology, Poznań University of Medical Sciences, Poznań, Poland

Dear Editor,

Dr. Stamfelj’s letter to Editor raises several concerns with the article “Analysis of human dentition from Early Bronze Age: 4000-year-old puzzle” by Przystańska et al.

We are extremely glad for the interesting comments. We are pleased that our research article has provided stimulus for further discussion.

We agree with Dr. Stamfelj that the ASUDAS [1] is used for scoring dental morphology. Dental morphology is shared by paleontologists, odontologists, dental anthropologists, geneticists and dentists [2]. Paleontologists have developed nomenclatures intended to impart phylogenetic information on homologies in crown structure, while dental anatomists and dentists use positional terms for major cusps and other features of the tooth crown that are unambiguous [2]. Although assessment of tooth morphology was the part of aim of our study, the paper focuses more on the pathologies and age determination. Our intention was to make it more familiar for wider group of readers, than only dental anthropologists; therefore, we decided to use anatomical nomenclature commonly used in dental clinical practice. This could have been explained in the paper and we are grateful to get the opportunity to clarify it in this short reply.

Dr. Stamfelj correctly pointed out that Fig. 2 provides information not harmonizing with the text. It presents actually maxillary, not mandibular permanent molar. We found this error undetected.

The author of the letter to the editor also presents his opinion on the root morphology of molars described in our study. Assessment of tooth morphology, especially when the corresponding alveolar socket (bony fragment) is lacking, is a complicated and demanding task, even for experienced specialist, and the observations are always burdened with uncertainty. Realizing the difficulties, the presented study was undertaken by a large interdisciplinary team including experienced anthropologists and odontologists, also specialized in human dental morphology and radiology. Therefore, the results are presented by consensus and were previously critically reviewed and discussed among the members of our team as well as researchers who had the opportunity to see the results during the thematic conferences.

Taking into account the narrow scientific community and vast number of articles published in the field, we appreciate the alternative interpretation of Dr. Stamfelj which extends the discussion.
